# Clinicians’ Interpretation of Unreported Chest Radiographs in Biologic Prescription Workup Service: A Comprehensive Analysis

**DOI:** 10.7759/cureus.48852

**Published:** 2023-11-15

**Authors:** Fatima Farman, Awin Mohammed Murad, Kehinde O Sunmboye

**Affiliations:** 1 General Internal Medicine, University Hospitals of Leicester NHS Trust, Leicester, GBR; 2 College of Health Sciences, University of Leicester, Leicester, GBR; 3 Rheumatology, University Hospitals of Leicester NHS Trust, Leicester, GBR

**Keywords:** certainty interpreting cxr among clinicians, service improvement, inadequate training of cxr interpretation, pre-biologic workup, interpretation skills of chest x-rays, clinicians’ confidence interpreting cxr

## Abstract

Clinicians without a radiology specialization face difficulties when they attempt to interpret chest X-rays (CXRs), a crucial and extensively utilized diagnostic tool that plays a fundamental role in the detection of pulmonary and cardiovascular disorders. This cross-sectional study assessed the confidence and competence of clinicians, including junior specialty trainees, higher specialty trainees, and specialist nurses, in interpreting CXRs before starting biological treatment. An online survey was used to collect data from clinicians in various healthcare settings, focusing on their experience, training, confidence levels, and CXR interpretation proficiency. The survey uncovered clinicians’ insufficient confidence in interpreting the pre-biological screening CXRs despite their clinical expertise. This uncertainty raises concerns about potential misinterpretations, affecting timely treatment decisions. A Kruskal-Wallis test indicated a significant difference between training levels required with a p-value of 0.001, rejecting the null hypothesis. Subsequently, a Dunn-Bonferroni test revealed that the higher specialty trainee-specialist nurse pair differed significantly, with the specialist nurse group requiring more training. This study highlighted the need for enhanced radiology education for clinicians involved in chest radiograph interpretation for pre-biological screening. Implementing a structured training program is essential to improve skills and ensure accurate interpretation of non-formally reported chest radiographs, ultimately enhancing patient outcomes and healthcare practices.

## Introduction

Accounting for 25% of all radiographic scans, chest X-ray (CXR) is an essential diagnostic tool to assess the airways, pulmonary parenchyma and vessels, mediastinum, heart, pleura, and chest wall, with an average of 236 CXRs per 1000 patients per year [1]. CXR is performed to investigate and monitor a wide spectrum of diseases, for instance, pneumonia, heart failure, or pneumothorax, investigating suspected lung cancer, tuberculosis (TB), or interstitial lung diseases [2].

Despite its extensive use in radiography, CXR interpretation remains a formidable challenge [3]. International studies among medical students and postgraduate doctors highlight insufficient radiological skills emphasizing the crucial role of radiology training [4]. As a general concept, thorough comprehension of thorax anatomy is a crucial element for precise CXR interpretation [3]. Standard CXRs are typically taken in posterior-anterior (PA) and left lateral positions for best image quality, as PA minimizes heart magnification and enhances spatial resolution. However, if patients cannot stand or bedside imaging is needed, anterior-posterior (AP) projections are used, which can magnify the heart and result in potential issues like poor positioning, inspiration, motion artifacts, overlap, and reduced image contrast [3]. In addition, technical issues such as overexposure, underexposure, projection, and normal variations can further complicate CXR interpretation [5]. Despite CXR interpretation skills being considered an important aspect of clinical medical training for medical students and junior doctors, studies elsewhere proved that postgraduate doctors’ chest radiograph interpretation is poor [6]. International studies also depicted that both medical interns and final-year medical students alike are largely limited in their ability to make radiological diagnoses of simple and commonplace situations [7].

Biologic and small-molecule medications have revolutionized the treatment approach for multiple systemic inflammatory diseases such as rheumatoid arthritis (RA) [8]. RA is a persistent systemic inflammatory condition primarily impacting the synovial joints [9]. In the early stages, RA affects small joints, proceeding to large joints and potentially affecting the skin, eyes, heart, kidneys, and lungs. In many cases, joint damage occurs, leading to the weakening of tendons and ligaments [10].

This study assessed the level of confidence of clinicians, including specialty trainees, core level trainees, and rheumatology specialist nurses, in interpreting CXRs prior to initiating biological treatment at a busy National Health Service (NHS) university teaching hospital. The aim is to identify areas for potential improvement in radiology teaching. There have been no prior studies exploring this specialist topic.

## Materials and methods

A cross-sectional study was carried out at a busy NHS university teaching hospital in question. The hospital has approximately 1,000 beds and provides the main accident and emergency service for its region in the East Midlands. This study was conducted between May 2023 and August 2023 encompassing three main departments: rheumatology, dermatology, and gastroenterology.

The study encompassed junior doctors at all levels (foundation year one and two, core level trainees, and specialty registrars) rotating through the three primary departments (rheumatology, dermatology, and gastroenterology), along with specialist nurses. Out of the 56 participants, all willingly took part and provided their informed consent after being briefed on the study’s purpose.

An online survey of seven questions was constructed by using Windows Forms (Microsoft Corporation, Redmond, WA), and the QR code was distributed to all doctors rotating within rheumatology, dermatology, and gastroenterology departments within the hospital as well as distributing the questionnaire via email. The objective was to evaluate participants’ knowledge of pre-biologic prescription workup and their confidence in interpreting CXR prior to commencing biologic treatment. The questionnaire did not incorporate clinical data or radiological images.

The questionnaire collected data on clinician grades, their familiarity with pre-biologic workup for rheumatic disease patients requiring biologics, their ability to identify contraindications for biologic treatment, and their confidence interpreting unreported CXR before commencing biologic therapies. Additionally, the survey inquired about clinician’s interest in receiving training on CXR interpretation. Notably, there were no specified time limits or scoring for questionnaire completion.

Statistical analysis

The data were organized in a spreadsheet using Microsoft Excel 2021 (Microsoft Corporation, Redmond, WA). In this study, an evaluation of the clinician’s grade as the independent variable was then compared to the dependent variables assessing clinician familiarity, confidence, and training requirements in interpreting CXR prior to initiating biological treatments. Results were considered to be statistically significant when p < 0.05 in all analyses [7]. Prior to this study, there was no prior knowledge concerning clinicians’ familiarity and confidence in interpreting CXR before starting biologic therapies in line with hospital guidelines. No existing studies were available for reference. Consequently, the formal sample size calculation was omitted during the planning stage. The actual participant count was determined by practical constraints. However, efforts were made to include as many participants as possible in the study. Data were categorized and depicted according to their types. Since the data exhibited a non-normal distribution, non-parametric methods were utilized [4]. Thus, the Kruskal-Wallis test was employed when comparing more than two groups with their responses [11].

## Results

**Figure 1:**
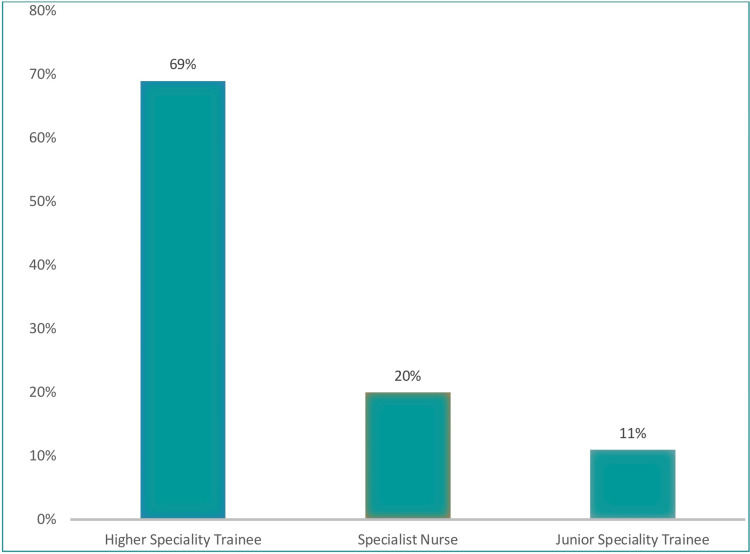
Grade of clinicians

Before analyzing the data, variable transformations were conducted and merged with specific variables. Clinician grades were subsequently divided into three groups: junior specialty trainees, higher specialty trainees, and specialist nurses. The first group comprised junior specialty trainees (consisting of first- and second-year trainees and core medical trainees), the second group comprised specialist nurses, and the third group was the higher specialist trainees (comprising trust grade doctors and specialist registrars) for comparative analysis (Figure 1).

**Figure 2:**
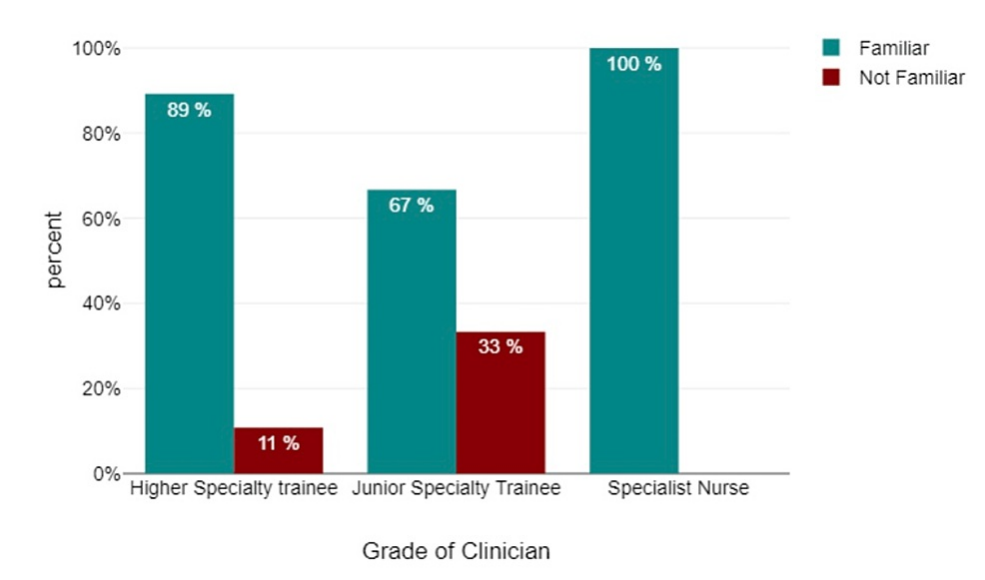
Are you familiar with pre-biologic workup for patients with rheumatic disease needing biologics?

In addition to the aforementioned data transformations, participant responses were consolidated by grouping those who reported "familiar" and "very familiar" with pre-biologic workup for patients with rheumatic diseases requiring biologics into a single category labeled "familiar." Similarly, responses from participants who indicated "neither familiar nor not familiar" and "not familiar and not very familiar" were merged to form the "not familiar" category (Figure 2). These transformations were implemented to enhance the clarity and interpret the ability of analysis.

**Figure 3:**
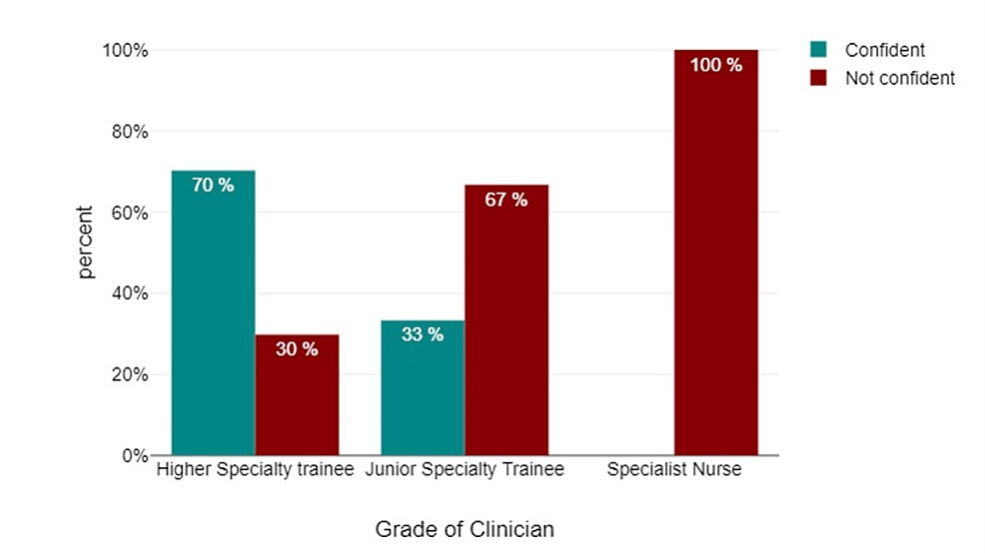
Do you feel confident interpreting chest X-rays prior to starting biological treatment?

**Figure 4:**
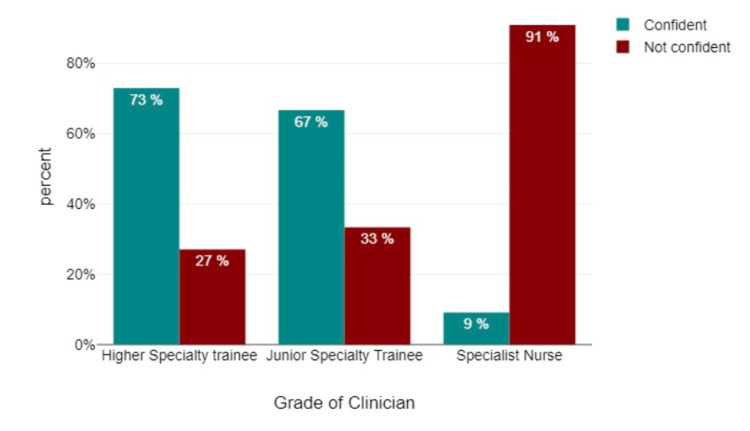
Do you feel confident in commencing on chest X-ray as a normal chest X-ray without a report?

Furthermore, there was a refining process of participant responses by grouping individuals who expressed "confident" and "very confident" levels of competence in identifying contraindications to biologic treatment based on medical history, interpreting CXRs before commencing biologic treatment (Figure 3), and providing comments on chest-X-rays without a formal report (Figure 4). This combined group was categorized as "confident." Correspondingly, participants who chose responses such as "not confident," "neither confident nor not very confident," and "not very confident" were integrated to establish the "not confident" category.

**Figure 5:**
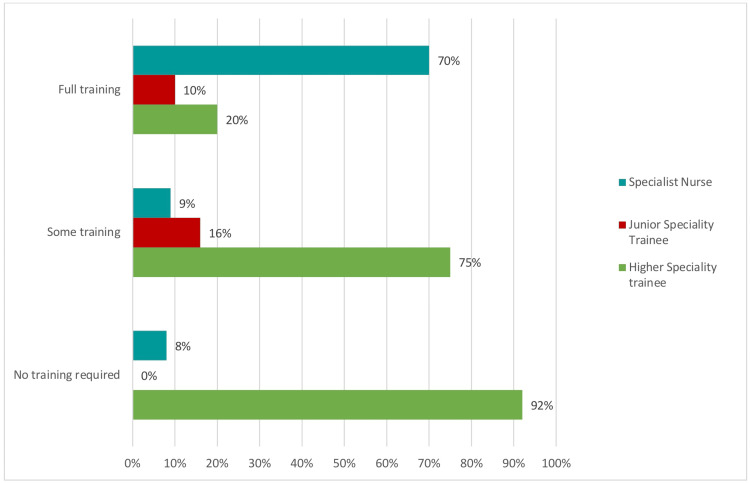
Level of training required

In relation to the training requirements, these responses were left in ordinal form from "no training required" to "some training required" to "full training required." These categories were left unchanged to facilitate methodological rigor and enhance the clarity of the analyzed data. With regards to the participants, a total of 56 responses were received, including 11% from junior specialty trainees, 20% from specialist nurses, and 69% from higher specialty trainees (Figure 5).

**Figure 6:**
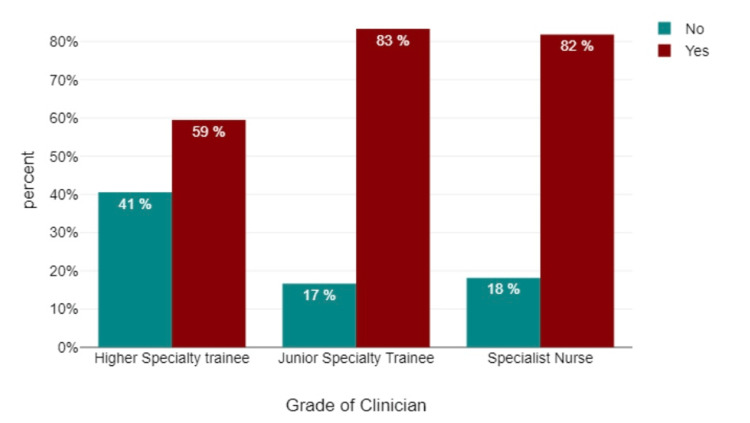
Do you feel you need more training in chest X-ray reporting?

Data analyses revealed that 78% of the participants exhibited familiarity with the pre-biological workup required for patients with rheumatic disease necessitating biologics, while 12.5% of participants indicated a lack of familiarity. Additionally, 62.5% of participants demonstrated confidence in recognizing contraindications to biologic treatment through medical history, whereas 37.5% did not. In terms of confidence in interpreting CXRs before initiating biological treatment, 57.7% of participants displayed confidence, while 48.2% did not. With regard to confidence in interpreting a CXR as "normal" without a formal report, 57.1% of participants displayed confidence, while 42.8% did not. When participants were surveyed regarding their need for CXR interpretation training, a significant majority (78.6%) of respondents expressed a clear desire for such training, while a smaller portion did not perceive it as necessary (Figure 6).

Explanation of the Kruskal-Wallis test

A Kruskal-Wallis test showed that there is a significant difference between the categories of the independent variable with respect to the dependent variable "What level of training do you think you need if you feel you need Chest X-ray training?" (Table 1). The p-value is 0.001. Thus, with the available data, the null hypothesis is rejected (Table 2).

**Table 1:** Ranks of the clinician groups for Kruskal-Wallis analysis

Groups	n	Median	Mean rank
Higher specialty trainee	37	2	23
Junior specialty trainee	6	2	32
Specialist nurse	11	3	40
Total	54	2	

**Table 2:** Kruskal-Wallis test of the groups

	Chi^2^	df	p
What level of training do you think you need if you feel you need chest X-ray training?	13	2	0.001

As the Kruskal-Wallis test showed that there was a significant difference between groups, a Dunn-Bonferroni test was used to compare the groups in pairs to find out which ones were significantly different. The Dunn-Bonferroni test showed that the pairwise group comparison of higher specialty trainee and specialist nurse has an adjusted p-value of less than 0.05, and thus, based on the available data, it can be assumed that the two groups are significantly different, and the specialist nurse group require more training than the higher specialty trainee group (Table 3).

**Table 3:** Dunn-Bonferroni test (post hoc test) as the Kruskal-Wallis tests were significant

Groups	Test statistic	Std. error	Std. test statistic	P	Adj. P
Higher specialty trainee – junior specialty trainee	-9	6	-1	0.144	0.432
Higher specialty trainee – specialist nurse	-17	5	-4	<0.001	0.001
Junior specialty trainee – specialist nurse	-8	7	-1	0.263	0.79

## Discussion

In the era of digital advancement, accurate CXR interpretation remains a diagnostic challenge [12]. This study addressed the question of whether clinicians of different grades were familiar with the pre-biologic screen and assessed their level of certainty in interpreting CXRs without a formal report prior to initiating biologic therapies. A cross-sectional study was carried out at Leicester Royal Infirmary. This study was conducted across rheumatology, dermatology, and gastroenterology departments via an online survey of seven questions. Data from 56 participants were collected and analyzed using the Kruskal-Wallis test, yielding significant Z and R values for all questions. Plain films remain integral in daily clinical practice and are expected to maintain their significance in the foreseeable future [5].

While most clinicians were acquainted with pre-biologic screening, this study revealed a wide range of confidence and certainty levels among clinicians when interpreting CXR without a formal report. One potential rationale for this variation is the lack of formal training and guidance. As Vincent et al. argued expecting clinicians to acquire these skills without support is unrealistic [13,14]. Another possible explanation is that an excessive reliance on radiologists might affect the confidence and certainty in CXR interpretation.

A study was conducted by Samuel and Shaffer explaining that merely 29% of medical schools require diagnostic radiology training [15], leading to the absence of specific CXR interpretation training in undergraduate medical education [16]. Additionally, despite the typical expectation that accuracy and confidence in chest radiograph interpretation would increase with seniority, a study in 2003 revealed a contrary trend, with overall skill levels remaining suboptimal [5]. The issue of inadequate radiological training is not confined to a single region; it is a national concern [5]. While some trusts do offer radiology departments, the emphasis on their importance remains insufficient [5]. This deficiency has a direct impact on the quality of service provided to the community.

Another study found that seniority had a positive impact on the level of confidence in CXR interpretation [2]. However, a separate study in the UK, conducted among final-year medical students, revealed that less than 25% of them were confident and accurate in interpreting CXRs. This low confidence was attributed to limited formal training (ranging from two to 42 hours with a median of 21 hours) [17]. The absence of formal accreditation for CXR interpretation within local medical training programs poses a genuine issue, potentially affecting the quality of CXR interpretation skills among future doctors. This, in turn, may lead to delays in patient management and poorly controlled disease symptoms, ultimately impacting the efficacy of therapies [2]. Another separate study indicated that subjects found it challenging to interpret normal plain radiographs [18].

Another study conducted among junior doctors revealed that only 30.3% of house officers were proficient in interpreting CXRs. This deficiency could be attributed to limited formal training opportunities or the lack of emphasis on chest radiograph competency within the competency scheme [7]. Inadequate CXR interpretation skills will be reflected in the competency in managing patients in the outpatient department, which makes the majority of patients reviews across three main departments: rheumatology, dermatology, and gastroenterology. This can result in prolonged waiting hours. In some studies, it was observed that the time between the onset of symptoms of conditions like RA and the initiation of medication in various countries, including the UK, ranged from six to 19 months [19].

This study showed that despite the majority of respondents being familiar with the pre-biologic and the majority of participants being higher specialty registrars, their confidence in interpreting CXR remained sub-optimum. This can be attributed to the absence of an adequate training program. The lack of confidence can be reflected in longer waiting hours and delays in starting biological therapies. This can reflect its effect on the quality of care delivered to patients and subsequent outcomes and response to biological treatment.

Furthermore, this study highlighted an important issue in this trust that according to the existing knowledge has not been assessed previously.

Potential solutions

Providing junior trainees and specialist nurses with sufficient training to robust their confidence in interpreting CXR [20]. In one of the studies, the importance of using PACS (picture archiving and communication system) rather than plain films was highlighted, owing to better resolution and the ability to modify the images by altering the grey scale to reduce the effect of technical factors such as incorrect exposure [5].

Periodic refresher courses for clinicians engaged in CXR reporting are crucial to maintaining their skill levels. These courses could be delivered through an accessible online training platform, providing clinicians interpreting CXRs with ongoing opportunities for skill enhancement.

Collaboration among different specialties is essential for CXR interpretation. Introducing multidisciplinary training programs has the potential to improve communication and cooperation between clinicians and radiologists.

Limitations of the study

Poor Response Rate

A major number of the collected data was from distributing the questionnaire via email. Although most of the online participants were aware of the purpose of this study, reluctance to reply remained an issue. Hence, the total online responses received comprised only 26.7% of the total participants.

Selection Bias

The other limitation of this study was the possibility of selection bias. This might be linked to the fact that those contributors with more experience and confidence completed the questionnaire.

Formal Training Programs for Chest X-rays Not Available

Formal training programs for CXRs are not available within the institution. However, it is recognized that individuals in advanced stages of training may have developed confidence through practical experience. This limitation restricts the generalizability of the findings.

## Conclusions

This study aimed to evaluate the competency of clinicians across various specialties, including both junior and advanced specialty trainees, as well as specialist nurses, in the interpretation of CXRs for pre-biologic screening.

The distinctive and noteworthy aspect of this research lies in adopting descriptive ordinal values, such as very confident, confident, neither confident nor not confident, not confident, and not very confident, in the original questionnaire, deviating from numerical values. Acknowledging the subjectivity and variation in terms like "confident" and "certainty" based on individual self-assessment, this methodology ensures more reliable results, genuinely reflecting clinicians' confidence levels. Identifying a lack of competency and certainty among clinicians tasked with pre-biologic screening and CXR interpretation, this study sheds light on the potential consequence of inadequate training and guidance. It emphasizes the urgency of addressing this issue, calling for enhanced competency through structured training programs among clinicians. This, in turn, holds the potential to improve patient outcomes and advance healthcare practices.
